# An Easy-to-Use Public Health-Driven Method (the Generalized Logistic Differential Equation Model) Accurately Simulated COVID-19 Epidemic in Wuhan and Correctly Determined the Early Warning Time

**DOI:** 10.3389/fpubh.2022.813860

**Published:** 2022-03-07

**Authors:** Zhuoyang Li, Shengnan Lin, Jia Rui, Yao Bai, Bin Deng, Qiuping Chen, Yuanzhao Zhu, Li Luo, Shanshan Yu, Weikang Liu, Shi Zhang, Yanhua Su, Benhua Zhao, Hao Zhang, Yi-Chen Chiang, Jianhua Liu, Kaiwei Luo, Tianmu Chen

**Affiliations:** ^1^State Key Laboratory of Molecular Vaccinology and Molecular Diagnostics, School of Public Health, Xiamen University, Xiamen, China; ^2^Department of Infection Disease Control and Prevention, Xi'an Center for Disease Prevention and Control, Xi'an, China; ^3^Université de Montpellier, Montpellier, France; ^4^CIRAD, Intertryp, Montpellier, France; ^5^IES, Université de Montpellier-CNRS, Montpellier, France; ^6^Yichang Center for Disease Control and Prevention, Yichang, China; ^7^Hunan Provincial Center for Disease Control and Prevention, Changsha, China

**Keywords:** infectious diseases, logistic differential equation model, generalized logistic differential equation model, curve fitting, goodness-of-fit

## Abstract

**Introduction:**

Modeling on infectious diseases is significant to facilitate public health policymaking. There are two main mathematical methods that can be used for the simulation of the epidemic and prediction of optimal early warning timing: the logistic differential equation (LDE) model and the more complex generalized logistic differential equation (GLDE) model. This study aimed to compare and analyze these two models.

**Methods:**

We collected data on (coronavirus disease 2019) COVID-19 and four other infectious diseases and classified the data into four categories: different transmission routes, different epidemic intensities, different time scales, and different regions, using *R*^2^ to compare and analyze the goodness-of-fit of LDE and GLDE models.

**Results:**

Both models fitted the epidemic curves well, and all results were statistically significant. The *R*^2^ test value of COVID-19 was 0.924 (*p* < 0.001) fitted by the GLDE model and 0.916 (*p* < 0.001) fitted by the LDE model. The *R*^2^ test value varied between 0.793 and 0.966 fitted by the GLDE model and varied between 0.594 and 0.922 fitted by the LDE model for diseases with different transmission routes. The *R*^2^ test values varied between 0.853 and 0.939 fitted by the GLDE model and varied from 0.687 to 0.769 fitted by the LDE model for diseases with different prevalence intensities. The *R*^2^ test value varied between 0.706 and 0.917 fitted by the GLDE model and varied between 0.410 and 0.898 fitted by the LDE model for diseases with different time scales. The GLDE model also performed better with nation-level data with the *R*^2^ test values between 0.897 and 0.970 vs. 0.731 and 0.953 that fitted by the LDE model. Both models could characterize the patterns of the epidemics well and calculate the acceleration weeks.

**Conclusion:**

The GLDE model provides more accurate goodness-of-fit to the data than the LDE model. The GLDE model is able to handle asymmetric data by introducing shape parameters that allow it to fit data with various distributions. The LDE model provides an earlier epidemic acceleration week than the GLDE model. We conclude that the GLDE model is more advantageous in asymmetric infectious disease data simulation.

## Introduction

Starting in December 2019, coronavirus disease 2019 (COVID-19) outbreak has spread widely around the world (1, 2). On March 11, the WHO declared COVID-19 as a global pandemic (3). Up to now, the total number of confirmed cases has reached 338,164,251, including 55,67,277 deaths, which causes a severe disease burden in many countries and regions around the world.

In addition to COVID-19, a variety of emerging infectious diseases in recent years, such as severe acute respiratory syndrome (SARS), influenza A virus subtype H1N1 (A/H1N1), Ebola virus disease (EVD), Middle East respiratory syndrome, and avian influenza, also pose serious threats to human health and life safety (4–6). Since the SARS outbreak in 2003, the government, research institutions, and public health departments have fully realized the importance of rapid identification and early intervention in infectious diseases (7, 8). In the following years, China's public health system has undergone a series of adjustments. Early prevention and control strategies of the epidemic were gradually improved through the implementation of various prevention and control measures, which have also greatly improved the prevention and control of infectious diseases in China (4, 9). However, with the rampant emergence of new infectious diseases such as COVID-19, the global infectious disease prevention and control situation remain grim and poses a serious threat to the global economy and national wellbeing (10).

Currently, the development of epidemic response strategies is usually based on traditional epidemiological and empirical methods. However, these methods are difficult to meet public health requirements, such as assessing the effectiveness of prevention and control measures, when there are significant uncertainties in the epidemiology of infectious diseases, including unknown or readily mutable epidemic pathogens, unclear transmission characteristics, long and complex pathogenesis, and low reproducibility in most cases. Therefore, when analyzing epidemics, we need an effective tool to repeatedly simulate the transmission routes of infectious diseases and to rapidly predict epidemics in different severity states for the purpose of prediction and early warning. In this context, the phenomenological model is particularly important for disease transmission modeling to estimate transmission potential at an early stage, predict the epidemic trajectory in the short term, and forecast the final epidemic size (11).

Since the end of the 20th century, mathematical models have been widely used in the reproduction of the new features of the dynamics of the epidemic, simulating, predicting, early warning, and the formulation of public health strategies (12–16), such as the logistic differential equation (LDE) model, autoregressive integrated moving average model, and transmission dynamic model. Due to the lack of strict mechanistic explanation of transmission and disease etiology (such as incubation period, latent period, and infectious period), the application of the logistic models in human epidemiology is not as extensive as that of transmission dynamic models such as susceptible-infective-removed (SIR) and susceptible-exposed-infective-asymptomatic-removed (SEIAR) models (1, 2, 17). However, its simplicity, low parameter requirements, and ability to visualize and rapidly reflect the prevalence of an epidemic have given it a place in the study of mechanistic models (18, 19), especially considering the urgency and severe consequence of the COVID-19 pandemic for public health management and decision-making. In the context of COVID-19, the LDE model was used to explain the epidemic (20–22). Studies have demonstrated that the LDE model can show differences in key disease dynamic parameters before and after interventions in China (17) and can determine when the rate of epidemic growth decreases (2). The generalized logistic differential equation (GLDE) model has also been used in practical applications (1, 21, 23–26). For example, studies have used the GLDE model to make simple predictions of the transmission potential and end time of the COVID-19 pandemic, which suggests it is a valuable tool for characterizing the transmission dynamics of COVID-19(19) and the effectiveness of interventions (27).

Our research team has previously applied the LDE model to quantify epidemics into early, medium, and late stages and to predict and warn of other infectious diseases such as hand, foot, and mouth disease (HFMD) (28), influenza A (H1N1) (28, 29), infectious diarrhea (30), and mumps (31). As mentioned in our research (32), although the results show good fitness, there were limitations. The LDE model requires data symmetry and is not suitable for long-term fitting. However, the actual data are asymmetric and the fitting curve is “narrower and taller” or “broader and shorter” than the actual curve when using the LDE model. The GLDE model adds shape parameters based on the LDE model to compensate for these drawbacks. Therefore, we intend to use COVID-19 data from Wuhan City to compare and analyze the advantages and disadvantages of these models in simulating infectious diseases and to generalize and validate them in other diseases.

## Materials and Methods

### Data Sources

We collected COVID-19 data. To further generalize and test the models, we also collected data on the other four diseases, which include influenza, mumps, HFMD, and acute hemorrhagic conjunctivitis (AHC). Transmission routes of these diseases were *via* respiratory, intestinal, and contact, respectively. Most of the data of incidence were collected from the National Health Commission of the People's Republic of China, and some were from published studies or WHO.

1) COVID-19: Daily COVID-19 data for Wuhan, China, from December 2, 2019 to March 16, 2020, were cited from a previous study by our team (33).
2) Other diseases: (A) Transmission *via* the respiratory tract: COVID-19, influenza, and mumps were used as examples in this paper. We collected the national-level influenza surveillance data published by WHO for China from the 1st week of 2001 to the 46th week of 2015. The influenza data included five subtypes: A(H1N1) seasonal, A(H1N1pdm09), A(H3N2), B (Victoria lineage), B (Yamagata lineage), and the sum of influenza A and B. We also collected national-level influenza surveillance data published by WHO for Argentina, Australia, China, Germany, South Africa, and the United States of America from the 1st week of 2015 to the 52nd week of 2019, and we used the total number of influenza-positive viruses. Daily data of mumps for Yichang City, from January 1, 2004 to December 31, 2016, were collected from the National Health Commission of the People's Republic of China.
b) Transmission *via* intestinal: We took the HFMD of two regions in China as an example. The weekly HFMD data from the 1st week of 2009 to the 52nd week of 2016 in Xi'an City, China were collected from the National Health Commission of the People's Republic of China. The weekly HFMD data from the 1st week of 2009 to the 52nd week of 2017 in Changsha City, China, were collected from our team's previous study (34).
c) Transmission *via* contact: AHC was taken as an example. The daily reported AHC data from September 1, 2010 to September 21, 2010 in Hunan Province, China, were collected from our team's previous study (35).

**Figure 1:**
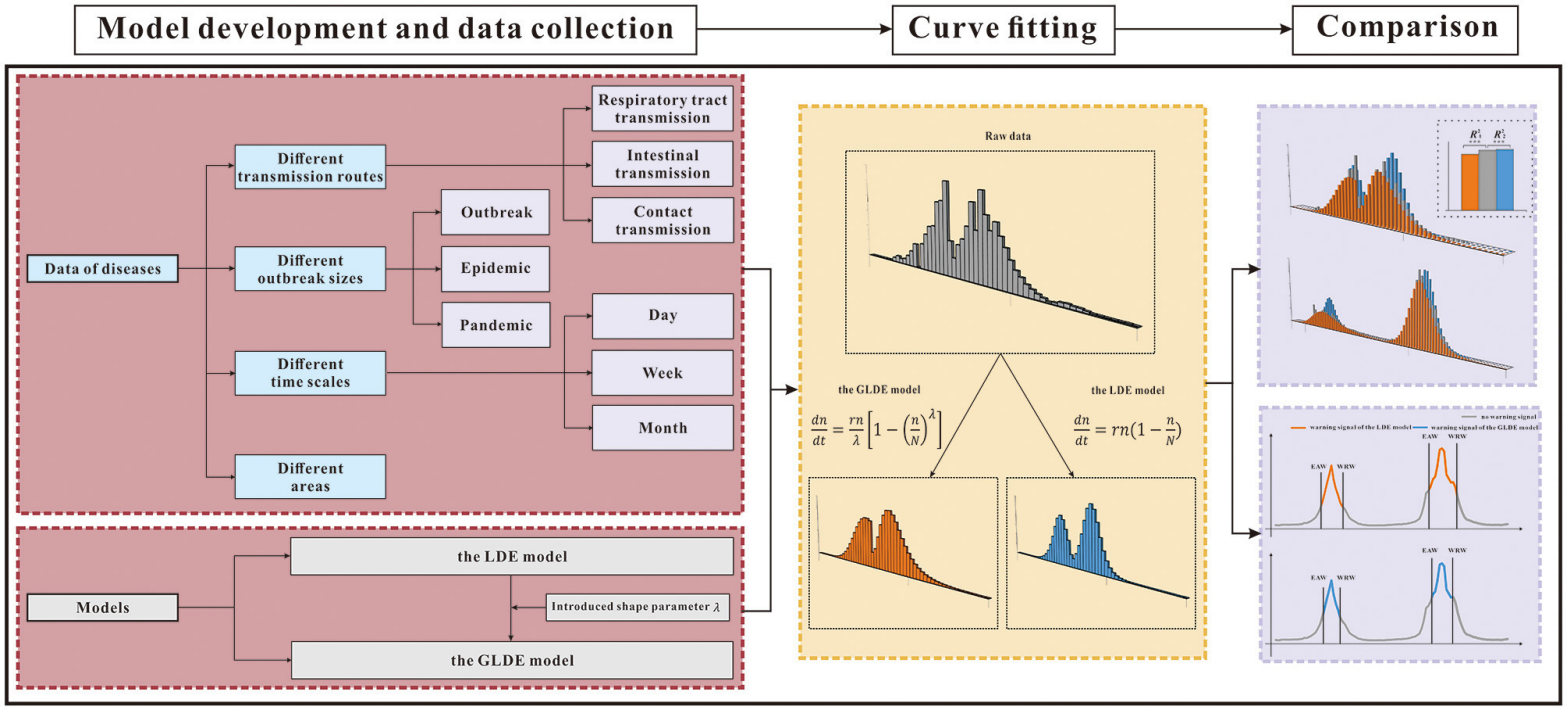
Research framework. GLDE, Generalized logistic differential equation; LDE, logistic differential equation; **p < 0.01; ***p < 0.001; EAW, epidemic accelerate week; WRW, warning remove week.

We compared and analyzed the goodness-of-fit and early warning of the LDE and GLDE models through COVID-19 data. To further generalize and test, we also classified other disease data into four categories from different perspectives: different transmission routes, different epidemic intensities, different time scales, and different areas to analyze the advantages and disadvantages of the models (Table 1). The research framework is shown in Figure 1.

**Table 1:** Disease types and areas at different levels.

**Group**		**Disease**	**Areas**
Different transmission routes	Respiratory	COVID-19	Wuhan City
		Influenza	China
	Intestinal	HFMD	Xi'an City and Changsha City
	Contact	AHC	Hunan Province
Different outbreak sizes	Outbreak	Influenza	A school of Changsha City
	Epidemic		Changsha City
	Pandemic		China
Different time scales	Day	Mumps	Yichang City
	Week		
	Month		
Different areas		Influenza	American, Argentina, Australia, China, Germany and South Africa

### Epidemic Intensity Definition Based on Scales of Cases

We classified the influenza data as three epidemic intensities: outbreak, epidemic, and pandemic according to the definition from The Centers for Disease Control and Prevention (CDC) (36).

Epidemic refers to an increase, often sudden, in the number of cases of a disease above what is normally expected in that population in that area.

Outbreak carries the same definition of epidemic but is often used for a more limited geographic area.

Pandemic refers to an epidemic that has spread over several countries or continents, usually affecting a large number of people.

### Model Development

#### The LDE Model

The LDE model was mainly used to describe population growth in the early stage and gradually was applied to the medical field (22). The differential equation is in Equation (1) (32), and its general solution is in Equation (2),


(1) $$\begin{array}{l} \frac{dn}{dt}=rn\left(1-\frac{n}{N}\right) \end{array}$$

(2) $$\begin{array}{l} n=\frac{N}{1+e^{-rt-c}} \end{array}$$

where *n* is the cumulative number of cases, *N* is the upper line of the cumulative cases, *r* is the growth rate coefficient, and *c* is the constant generated when solving the LDE. dn/dt is the ratio of cumulative cases to time, which represents the change rate of cumulative cases *n* of infectious diseases at time *t*, and can also represent the model characteristics of new cases over time.

This model has three parameters, *r, n*, and *N*. The parameter values of *r* and *N* determine the specific shape of the model curve, and accurate parameter estimation will greatly improve fitting. According to the initial and critical conditions set, the critical value *N* at both ends of the LDE is known, and only the parameter *r* is unknown. However, in practical applications, the value of *N* is difficult to obtain because of the limitation of conditions; therefore, parameter estimation is sometimes required.

As the disease develops and immune barriers are built up in the population, the epidemic gradually reaches a stable state and the number of new cases gradually decreases until the epidemic is over. Therefore, the logistic model curve presents a “slow–fast–slow” trend, which shows an *S* shape. The point where the LDE model curve changes from slow to fast is called the inflection point. Doing the third-order derivation over Equation (2) to make the new equation equal to zero, and the result is Equation (3), whereas *t*_1_ in Equation (4) is the inflection point of the curve of the LDE model, that is epidemic accelerate week.


(3) $$\begin{array}{l} t=-c\pm 1.317/r \end{array}$$

(4) $$\begin{array}{l} t_{1}=-c-1.317/r \end{array}$$

#### The GLDE Model

The LDE model requires data to be symmetrical, but data in some cases do not meet this requirement. Half a century ago, one classical logistic model was extended to allow S-shape to have more flexible curvature in the case of the asymmetric growth curve, so as to establish Richards' curve, that is, the GLDE model (37). After applying four parameters to the LDE model, there were five types of the GLDE model. The GLDE model with shape parameters can fit case data of various distribution types, so of interest in this study was the type of shape parameter introduced. The differential equation is in Equation (5), and the general solution is in Equation (6).


(5) $$\begin{array}{l} \frac{dn}{dt}=\frac{rn}{\lambda }\left[1-{\left(\frac{n}{N}\right)}^{\lambda }\right] \end{array}$$

(6) $$\begin{array}{l} n=\frac{N}{{\left(1+e^{-rt+c}\right)}^{\frac{1}{\lambda }}}\left(N,\lambda ,r\rangle 0\right) \end{array}$$

By calculating the first and second derivatives of the general solution of the GLDE and letting it be 0, we can obtain the unique inflection point. The growth rate of the GLDE model curve decreases with a decrease in *nt*, and the rate of decrease is related to the shape parameter λ. At first, the curve shape is concave, and the growth rate accelerates; after the inflection point, the curve is convex, the growth rate declines and finally tends to saturation. The GLDE model curve has four parameter values, *r, n, N*, and λ, and the shape of the curve is determined by the values of *r, N*, and λ.

As can be seen from the equation, when the shape parameters fall within the range of (0,1), the distribution is skewed to the left; when they are >1, the distribution is skewed to the right, and when they are equal to 1, the graph is symmetrically distributed, that is, the general logistics distribution.

Doing the third-order derivation over Equation (6) to make the new equation equal to zero, and the result is in Equation (7), whereas *t*_1_ in Equation (8) is the inflection point of the curve of the GLDE model, that is epidemic accelerate week.


(7) $$\begin{array}{l} t=-\frac{c-\ln\left(\frac{3\pm \sqrt{5}}{2}\lambda \right)}{r} \end{array}$$

(8) $$\begin{array}{l} t_{1}=-\frac{c-\ln\left(\frac{3-\sqrt{5}}{2}\lambda \right)}{r} \end{array}$$

### Parameter Estimation

As shown in Table 2, there were five parameters, where *r* is the growth rate coefficient, *n* is the cumulative number of cases, *N* is the upper line of the cumulative cases, λ is the shape parameter, and *c* is the constant generated when solving the differential equations.

**Table 2:** Definitions and sources of model parameter.

**Parameter**	**Description**	**Value**	**Range**	**Method**
*r*	Growth rate coefficient	-	≥0	Model fitting
*n*	Cumulative number of cases	-	≥0	Actual data
*N*	Upper line of the cumulative cases	-	≥0	Model fitting
λ	Shape parameter	-	≥0	Model fitting
*c*	Constant	-	≥0	Solving differential equations

The corresponding parameters *r, N*, and λ are obtained by model fitting (see Supplementary File 1).

### Model Fitting

Model fitting was performed on the number of cases. From the plots of the LDE model, the epidemic data in accordance with the LDE model, the plot of the number of cases over time should have a single-wave shape. Therefore, before fitting, the data must be divided into different segments, each consisting of a wave. The division of the data cycle could be determined according to the transmission characteristics of the diseases. For diseases with an obvious time cycle, it could be segmented according to their epidemic cycles. However, most infectious diseases did not have a specific cycle, or the cycle was not constant over time due to changes in influencing factors. Therefore, it was difficult to segment data according to a certain period of time.

Therefore, based on the assumptions of the LDE model and the characteristics of the curves, we segmented the period during the epidemic season (the interval between the first trough and the next trough). When two peaks with large intervals or fine tail segments appear, we removed these segments and fit only the peaks of the data. For data where peaks had bifurcations, we could choose whether to perform segmentation based on the size of the bifurcation. We compared the differences in the results by fitting the disease data of different disease types, different epidemic intensities, different time scales, and different areas to verify whether the two models can be used to fit the data of different types of epidemics and to explore their respective applicable conditions when dividing the “slow–fast–slow” period for infectious disease progression.

### Epidemic Accelerate Time Prediction

The epidemic accelerate weeks of influenza data in each country were calculated by Equations (5) and (8), respectively. The epidemic accelerate time in 2019 was predicted according to the corresponding median from 2015 to 2018 and then compared to the reality to analyze the predicting effects.

### Statistical Methods

Berkeley Madonna 8.3.18 software was used in the simulation of these models. Microsoft Excel 2019 software (Microsoft Corp, USA) was used for entering and managing related data and related mapping. The differential equation solving method was used with a fourth-order Runge-Kutta method with a tolerance of 0.001. The model convergence method was the least root mean square (LRMS) of the simulated data and the actual data. IBM SPSS Statistics for Windows, version 21.0 was used to calculate the coefficient of determination (*R*^2^) and *p*-values as the criterion to analyze and compare the goodness-of-fit of curves,


(9) $$\begin{array}{l} R^{2}=\frac{S_{y^{\prime }}^{2}}{S_{y}^{2}} \end{array}$$

where the ${\text{S}}_{{\text{y}}^{\text{′}}}^{2}$is the variance of the fitted value, the ${\text{S}}_{\text{y}}^{2}$ is the variance of the actual value. When *p* < 0.05, the closer *R*^2^ was to 1, the better the goodness-of-fit of the model. The example data and Berkeley Madonna code are given in Supplementary Files 2, 3.

## Results

**Figure 2:**
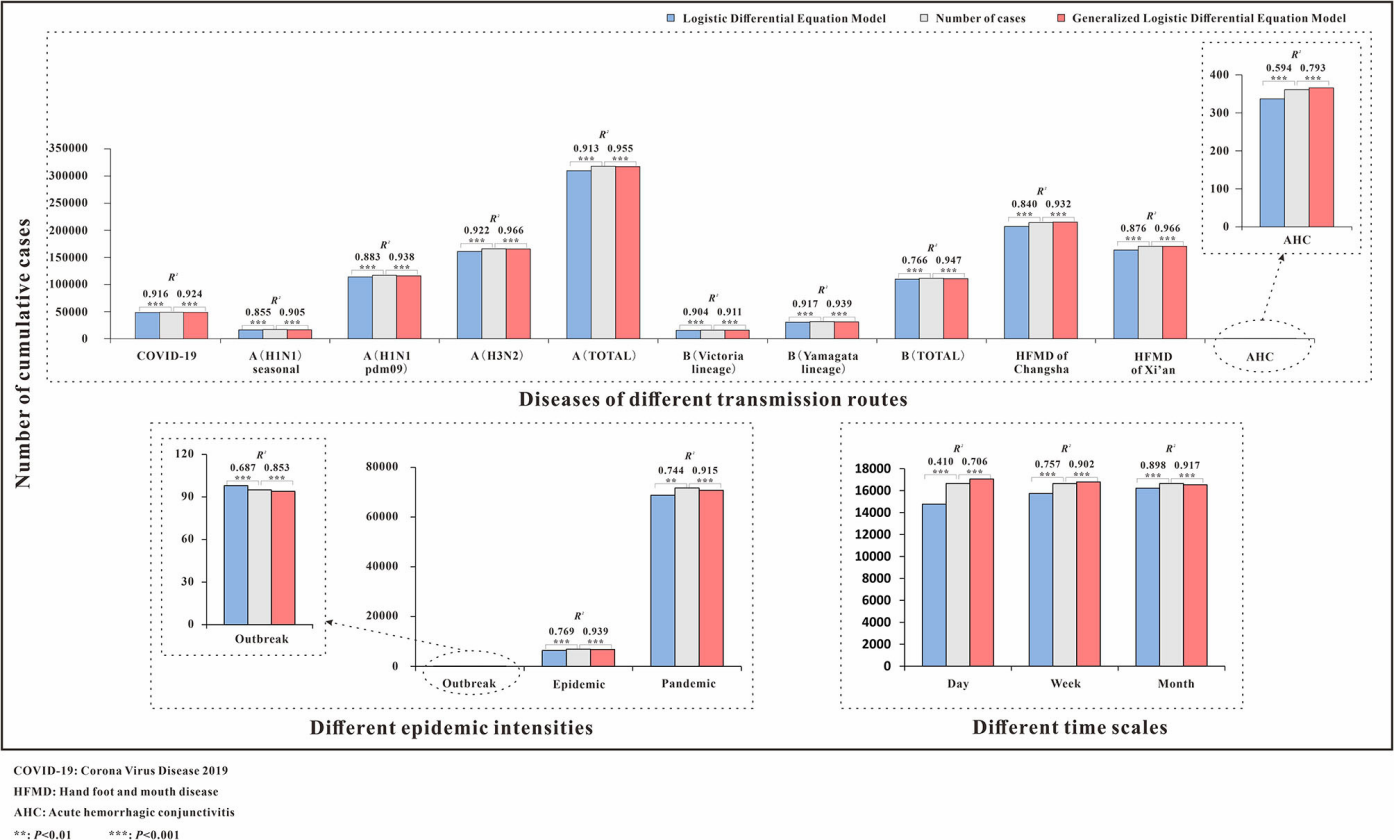
Number of cumulative cases and *R*^2^ of logistic and generalized logistic differential equation models. COVID-19, coronavirus disease 2019; HFMD, hand, foot, and mouth disease; AHC, acute hemorrhagic conjunctivitis; **p < 0.01; ***p < 0.001.

The fitting graphs showed that both models fitted the case data well, and the GLDE model was more suitable for the actual data. We performed LRMS analysis on the results of the two models separately and found that all results were statistically significant (*p* < 0.05). However, the *R*^2^ calculated using the GLDE model to fit the data was higher (Table 3, Figure 2).

**Table 3:** The results of goodness-of-fit.

		**LDE**		**GLDE**	
		***R^**2**^***	***P***	***R^**2**^***	***P***
COVID-19		0.916	<0.001	0.924	<0.001
Influenza A	H1N1 seasonal	0.855	<0.001	0.905	<0.001
	H1N1pdm09	0.883	<0.001	0.938	<0.001
	H3N2	0.922	<0.001	0.966	<0.001
	Total	0.913	<0.001	0.955	<0.001
Influenza B	Victoria lineage	0.904	<0.001	0.911	<0.001
	Yamagata lineage	0.917	<0.001	0.939	<0.001
	Total	0.766	<0.001	0.947	<0.001
Hand, foot and mouth disease	Changsha	0.840	<0.001	0.932	<0.001
	Xi'an	0.876	<0.001	0.966	<0.001
Acute hemorrhagic conjunctivitis		0.594	<0.001	0.793	<0.001
Outbreak		0.687	<0.001	0.853	<0.001
Epidemic		0.769	<0.001	0.939	<0.001
Pandemic		0.744	0.001	0.915	<0.001
Day		0.410	<0.001	0.706	<0.001
Week		0.757	<0.001	0.902	<0.001
Month		0.898	<0.001	0.917	<0.001
Influenza	American	0.953	<0.001	0.970	<0.001
	Argentina	0.900	<0.001	0.942	<0.001
	Australia	0.731	<0.001	0.922	<0.001
	China	0.846	<0.001	0.897	<0.001
	Germany	0.869	<0.001	0.931	<0.001
	South Africa	0.799	<0.001	0.970	<0.001

## COVID-19

**Figure 3:**
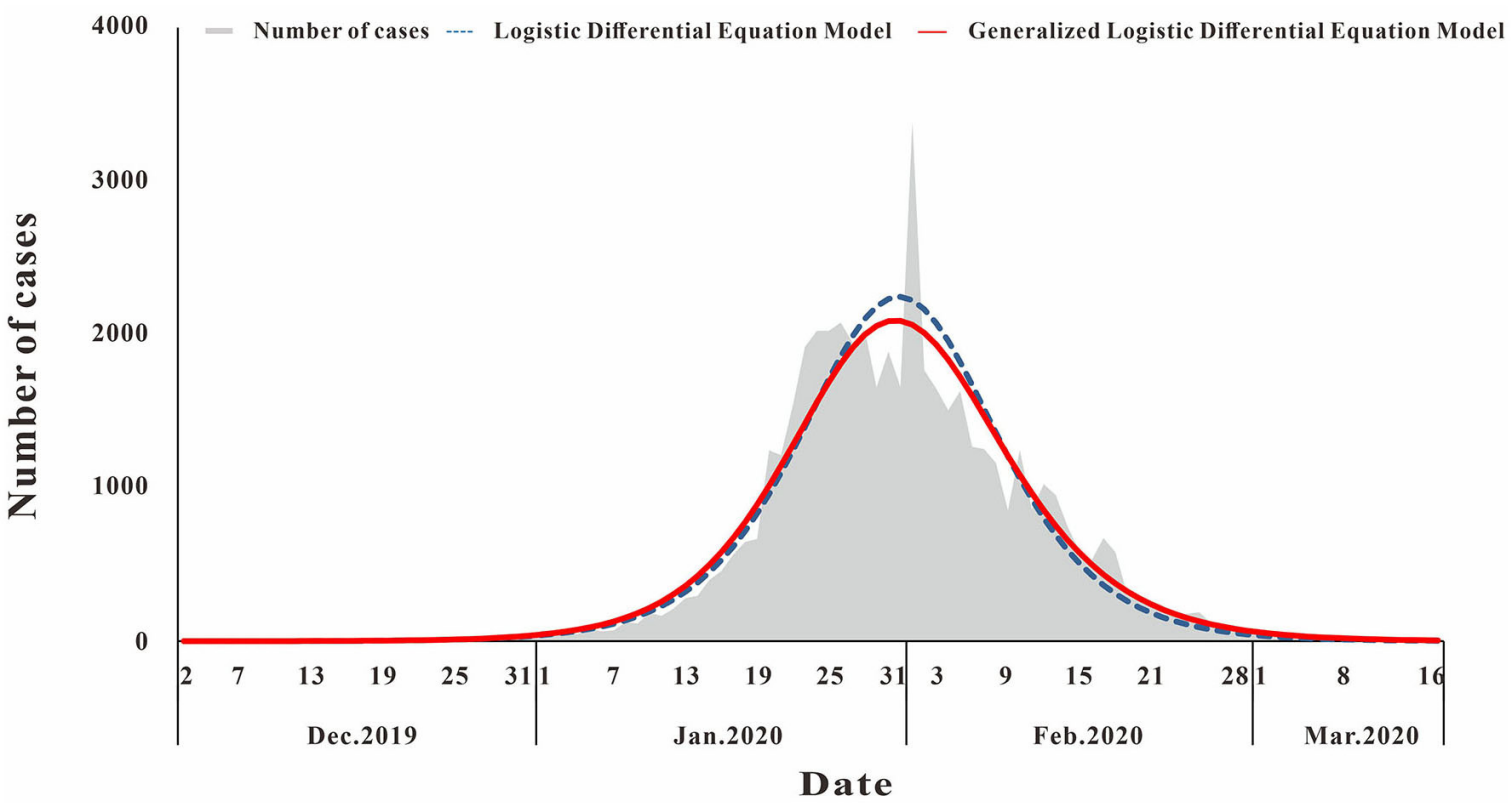
Curve fitting results of logistic and generalized logistic differential equation models of COVID-19.

The fitting diagram of COVID-19 in Wuhan city of the two methods is shown in Figure 3. The first wave of the COVID-19 pandemic in Wuhan lasted 106 days, and the cases showed an overall single-peak distribution so the data were not segmented. The number of cases began to decline after January 26, and there was an outlier on February 1. The *R*^2^ test value fitted by the LDE model was 0.916 (*p* < 0.001) and the *R*^2^ test value fitted by the GLDE model was 0.905 (*p* < 0.001).

According to Equations 5, 8, the epidemic acceleration time of COVID-19 in Wuhan calculated by the LDE model and the GLDE model was January 23 and January 24, respectively, and the LDE model was slightly earlier than the GLDE model.

## Other Diseases

### Infectious Diseases With Different Transmission Routes

**Figure 4:**
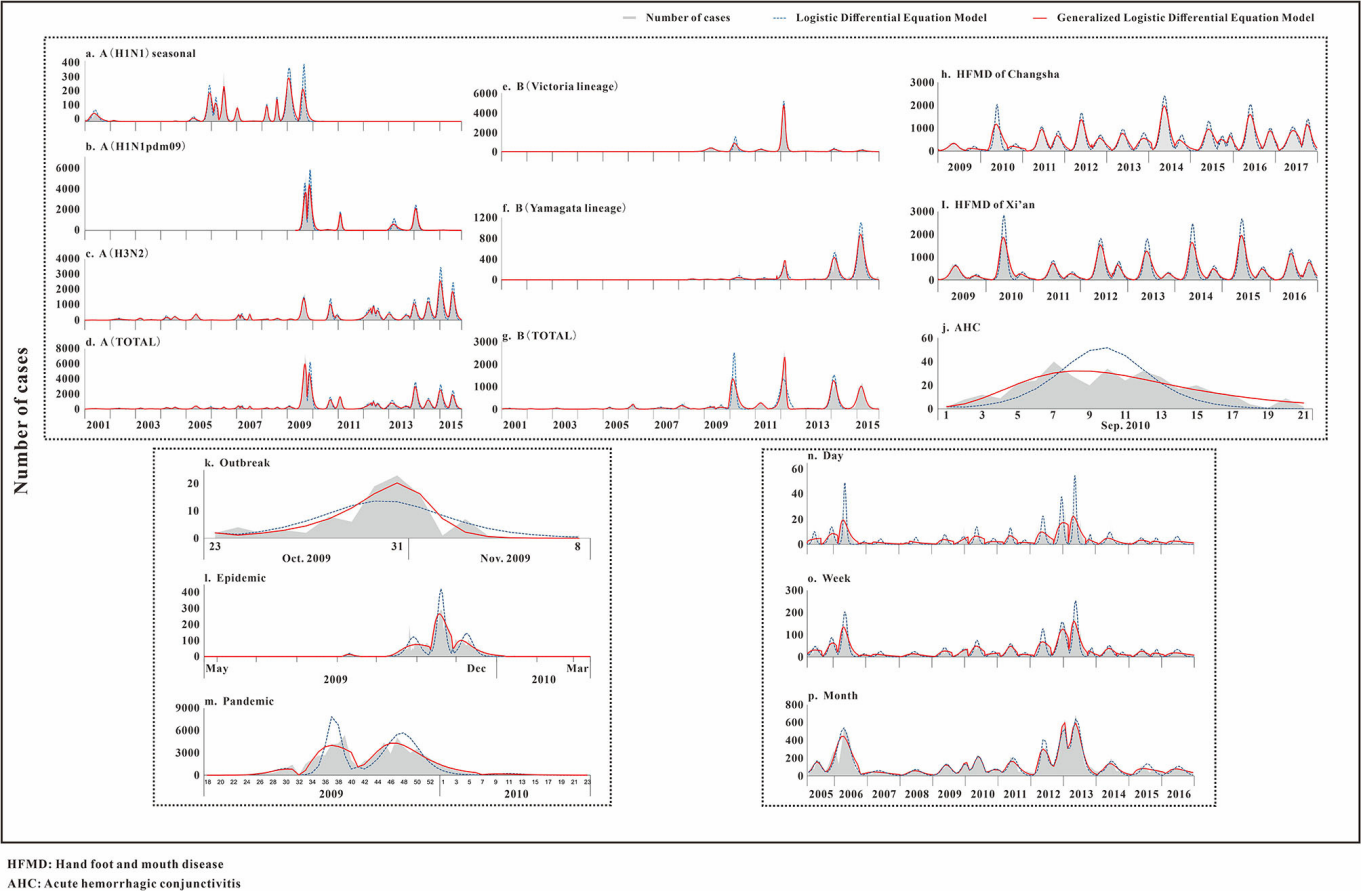
Curve fitting results of logistic and generalized logistic differential equation models. **(a)** A (H1N1) seasonal, **(b)** A (H1N1pdm09), **(c)** A (H3N2), **(d)** A (Total), **(e)** B (Victoria lineage), **(f)** B (Yamagata lineage), **(g)** B (Total), **(h)** HFMD of Changsha, **(i)** HFMD of Xi'an, **(j)** AHC, **(k)** Outbreak, **(l)** Epidemic, **(m)** Pandemic, **(n)** Day, **(o)** Week, and **(p)** Month. HFMD, Hand foot and mouth disease; AHC, Acute hemorrhagic conjunctivitis.

The fitting diagram of A (H1N1) seasonal, A (H1N1pdm09), A (H3N2), and the total number of influenza A infections among respiratory infectious diseases are shown in Figures 4a–d. The figure showed how disease peaks of different subtypes of influenza differed. The A (H1N1) seasonal data from the 48th week of 2010 to the 46th week of 2015 were 0. After abandoning these data, the remaining data were divided into 14 segments according to the epidemic curve. There were obvious fluctuations in the two peaks in 2009 and 2010. The epidemic curve showed that there were more cases in 2001 and 2005 than in 2009. The highest number of cases per week was 355, and it appeared in week 8 of 2009. The *R*^2^ test value fitted by the LDE model was 0.855 (*p* < 0.001), and the *R*^2^ test value fitted by the GLDE model was 0.905 (*p* < 0.001). In the influenza A (H1N1pdm09) data, the data from the 1st week of 2001 to the 17th week of 2009 were all zero. Therefore, the fitting data starting from the 18th week of 2009 were divided into eight segments. The epidemic curve showed that the number of cases in 2009 reached maximum, with an obvious wave peak. The highest number of cases per week reached 5,383, which occurred in the 39th week of 2009. At other times, the number of cases was low and sporadic. The *R*^2^ test value fitted by the LDE was 0.883 (*p* < 0.001), and the *R*^2^ test value fitted by the GLDE was 0.938 (*p* < 0.001). The distribution of influenza A (H3N2) data showed that it could be divided into 26 segments. The peaks in 2007 and 2012 were different from the traditional peaks, and they were fluctuated, with a small fluctuation at the peak. In 2001, from 36 weeks in 2005 to 36 weeks in 2006, and from 14 to 27 weeks in 2011, the number of cases was relatively small, and the wave peak was not distinct. The highest number of cases per week was 2,747, which occurred in the fourth week of 2015. The *R*^2^ test value fitted by the LDE model was 0.922 (*p* < 0.001), and the *R*^2^ test value fitted by the GLDE model was 0.966 (*p* < 0.001). The distribution map of the influenza A summary data showed that it could be divided into 28 segments. The number of cases after the 28th week of 2009 increased significantly compared with before, and the peak in 2012 was more flat; the highest number of cases per week was 7,280, which occurred in the 37th week of 2009. The *R*^2^ test value fitted by the LDE model was 0.913 (*p* < 0.001), and the *R*^2^ test value fitted by the GLDE model was 0.955 (*p* < 0.001).

The fitting graphs of B (Victoria lineage), B (Yamagata lineage), and the total number of B types are shown in Figures 4e–g. B (Victoria lineage) influenza data, which could be divided into eight segments according to the epidemic curve, showed that cases began to appear in the 20th week of 2008. The highest number of weekly cases reached 1,041, which occurred in week 8 of 2012. The *R*^2^ test value fitted by the LDE model was 0.904 (*p* < 0.001), and the *R*^2^ test value fitted by the GLDE model was 0.911 (*p* < 0.001). The B (Yamagata lineage) influenza data, which could be divided into 12 segments according to the epidemic curve, with four obvious peaks, showed that the cases appeared in week 20 of 2008. Among them, the 2014 and 2015 peaks were different from the traditional peaks, and all were serrated, that was, had two peak values. The highest number of cases per week reached 1,055, which occurred in week 10 of 2015. The *R*^2^ test value fitted by the LDE model was 0.917 (*p* < 0.001), and the *R*^2^ test value fitted by the GLDE model was 0.939 (*p* < 0.001). The distribution map of the influenza B summary data showed that it could be divided into 17 segments, among which four relatively dense peak groups appeared after 2010. In contrast to the traditional wave peaks, they were serrated. The highest number of cases per week was 2,630, which occurred in week 8 of 2012. The *R*^2^ test value fitted by the LDE model was 0.766 (*p* < 0.001), and the *R*^2^ test value fitted by the GLDE model was 0.947 (*p* < 0.001).

The fitting diagrams of HFMD in Xi'an city and Changsha City among enteric diseases are shown in Figures 4h,i, and the epidemic curves showed that the two cities annually presented double seasonal peaks. The epidemic curve of HFMD in Xi'an City could be divided into 14 segments. From 2009 to 2015, the peak in the first half was significantly higher than in the second, and the two peaks in 2016 were not significantly different. The highest number of cases per week reached 2,013, which occurred in week 23 of 2015. The *R*^2^ test value fitted by the LDE model was 0.876 (*p* < 0.001), and the *R*^2^ test value fitted by the GLDE was 0.966 (*p* < 0.001). The epidemic curve of HFMD in Changsha City could be divided into 19 segments. The distribution in 2015 differed from other years, not being an obvious bimodal distribution. Moreover, the first peaks in 2010, 2014, and 2016 differed from the traditional wave peak, all being serrated. The highest number of cases per week reached 2,163, which occurred in week 17 of 2014. The *R*^2^ test value fitted by the LDE model was 0.840 (*p* < 0.001), and the *R*^2^ test value fitted by the GLDE model was 0.932 (*p* < 0.001).

The fitting diagram of AHC in contact infectious diseases is shown in Figure 4j, and the data were not segmented. The *R*^2^ test value fitted by the LDE model was 0.594 (*p* < 0.001), and the *R*^2^ test value fitted by the GLDE model was 0.793 (*p* < 0.001).

### Infectious Diseases With Different Epidemic Intensities

The fitting graphs of the outbreak, epidemic, and pandemic are shown in Figures 4k–m. The outbreak data fitting results showed that the goodness-of- fit of the GLDE model (*R*^2^ = 0.853, *p* < 0.001) was better than that of the LDE model (*R*^2^ = 0.687, *p* < 0.001). The epidemic data were fitted with the flu data of Changsha City as an example, and the epidemic curve could be divided into seven segments. The *R*^2^ test value fitted by the LDE model was 0.769 (*p* < 0.001), and the *R*^2^ test value fitted by the GLDE was 0.939 (*p* < 0.001). The pandemic data contained the 2009 and 2010 influenza data of China as an example, and the epidemic curve could be divided into four segments. The *R*^2^ test value fitted by the LDE model was 0.744 (*p* < 0.001), and the *R*^2^ test value fitted by the GLDE model was 0.915 (*p* < 0.001).

### Infectious Disease From Different Time Scales

We used two methods to fit the mumps data from Yichang City with time scales of days, weeks, and months. The results are shown in Figures 4n–p. When taking days as the time scale, it could be divided into 21 segments according to the epidemic curve. Among them, there were more cases in 2006 and 2013 with the highest number of daily cases being 56, which occurred on 13 May 2013. The *R*^2^ test value fitted by the LDE model was 0.410 (*p* < 0.001), and the *R*^2^ test value fitted by the GLDE model was 0.706 (*p* < 0.001). When taking weeks as the time scale, it could be divided into 21 segments according to the epidemic curve. The highest number of weekly cases was 208, which occurred in week 19 of 2013. The *R*^2^ test value fitted by the LDE model was 0.757 (*p* < 0.001), and the *R*^2^ test value fitted by the GLDE model was 0.902 (*p* < 0.001). When the month was taken as the time scale, it could be divided into 15 segments according to the epidemic curve. The highest number of monthly cases was 686, which occurred in May 2013. The *R*^2^ test value fitted by the LDE model was 0.898 (*p* < 0.001), and the *R*^2^ test value fitted by the GLDE model was 0.917 (*p* < 0.001).

### Infectious Diseases in Different Areas

**Figure 5:**
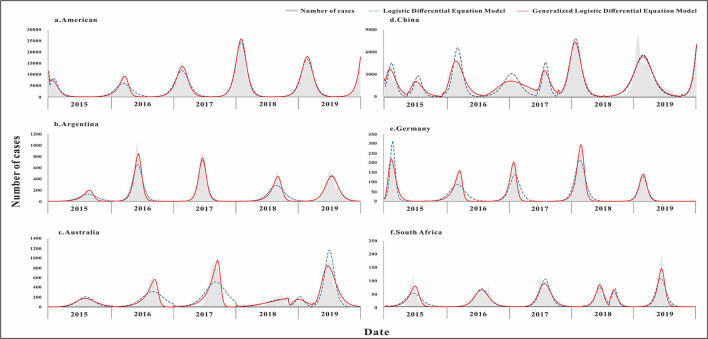
Curve fitting results of logistic and generalized logistic differential equation models in different areas. **(a)** American, **(b)** Argentina, **(c)** Australia, **(d)** China, **(e)** Germany, and **(f)** South Africa.

The fitting diagram of influenza in each country is shown in Figure 5. Influenza data in Argentina were divided into 5 segments according to the epidemic curve. The highest number of weekly cases was 1,072, which occurred in week 22 of 2016. The *R*^2^ test value fitted by the LDE model was 0.9 (*p* < 0.001) and the *R*^2^ test value fitted by the GLDE model was 0.942 (*p* < 0.001). Influenza data in Australia were divided into 6 segments according to the epidemic curve. The highest number of weekly cases was 1,002, which occurred in week 26 of 2019. The *R*^2^ test value fitted by the LDE model was 0.731 (*p* < 0.001) and the *R*^2^ test value fitted by the GLDE model was 0.922 (*p* < 0.001). Influenza data in China were divided into 8 segments according to the epidemic curve. The highest number of weekly cases was 8,426, which occurred in week 3 of 2019. The *R*^2^ test value fitted by the LDE model was 0.846 (*p* < 0.001), and the *R*^2^ test value fitted by the GLDE model was 0.897 (*p* < 0.001). Influenza data in Germany were divided into 5 segments according to the epidemic curve. The highest number of weekly cases was 292, which occurred in week 9 of 2018. The *R*^2^ test value fitted by the LDE model was 0.869 (*p* < 0.001), and the *R*^2^ test value fitted by the GLDE model was 0.931 (*p* < 0.001). Influenza data in South Africa were divided into 6 segments according to the epidemic curve. The highest number of weekly cases was 197, which occurred in week 24 of 2019. The *R*^2^ test value fitted by the LDE model was 0.799 (*p* < 0.001) and the *R*^2^ test value fitted by the GLDE model was 0.875 (*p* < 0.001). Influenza data in America were divided into 6 segments according to the epidemic curve. The highest number of weekly cases was 26,386, which occurred in week 5 of 2018. The *R*^2^ test value fitted by the LDE model was 0.953 (*p* < 0.001), and the *R*^2^ test value fitted by the GLDE model was 0.97 (*p* < 0.001).

**Figure 6:**
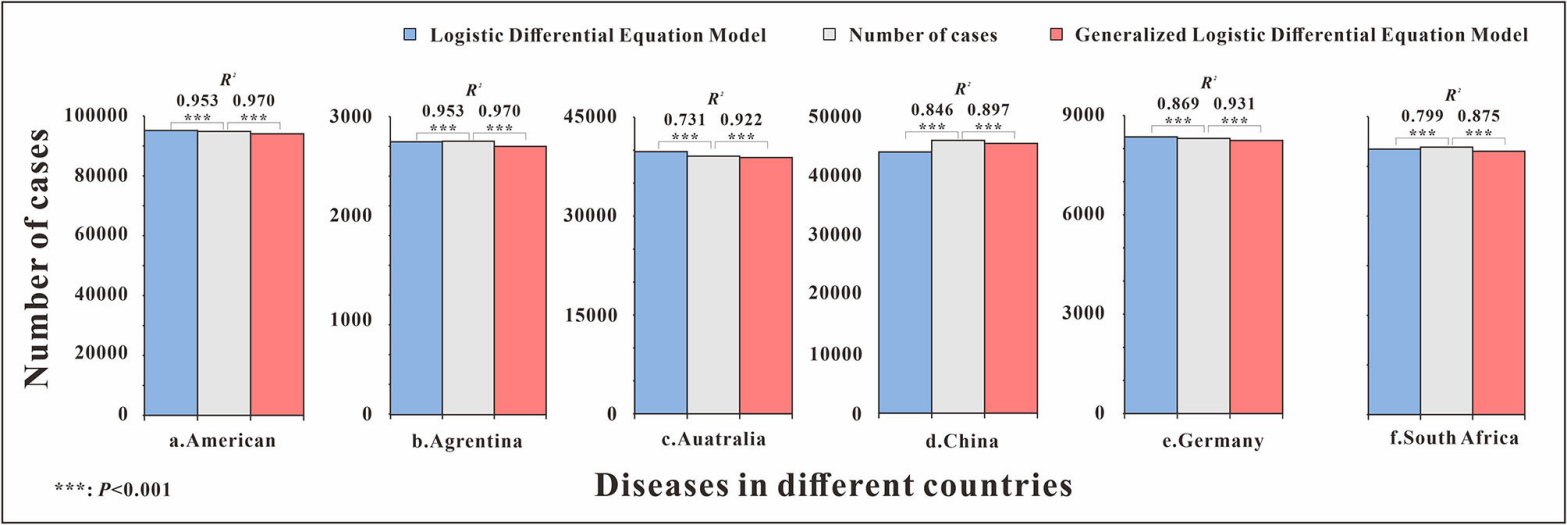
Number of cumulative cases and *R*^2^ of logistic and generalized logistic differential equation models in different areas. ***p < 0.001.

The results showed that the goodness-of-fit of the GLDE model was all better than that of the LDE model in each country, and the cumulative cases and the *R*^2^ values of the goodness-of-fit test are shown in Figure 6.

### Determination of the Early Warning Week

**Figure 7:**
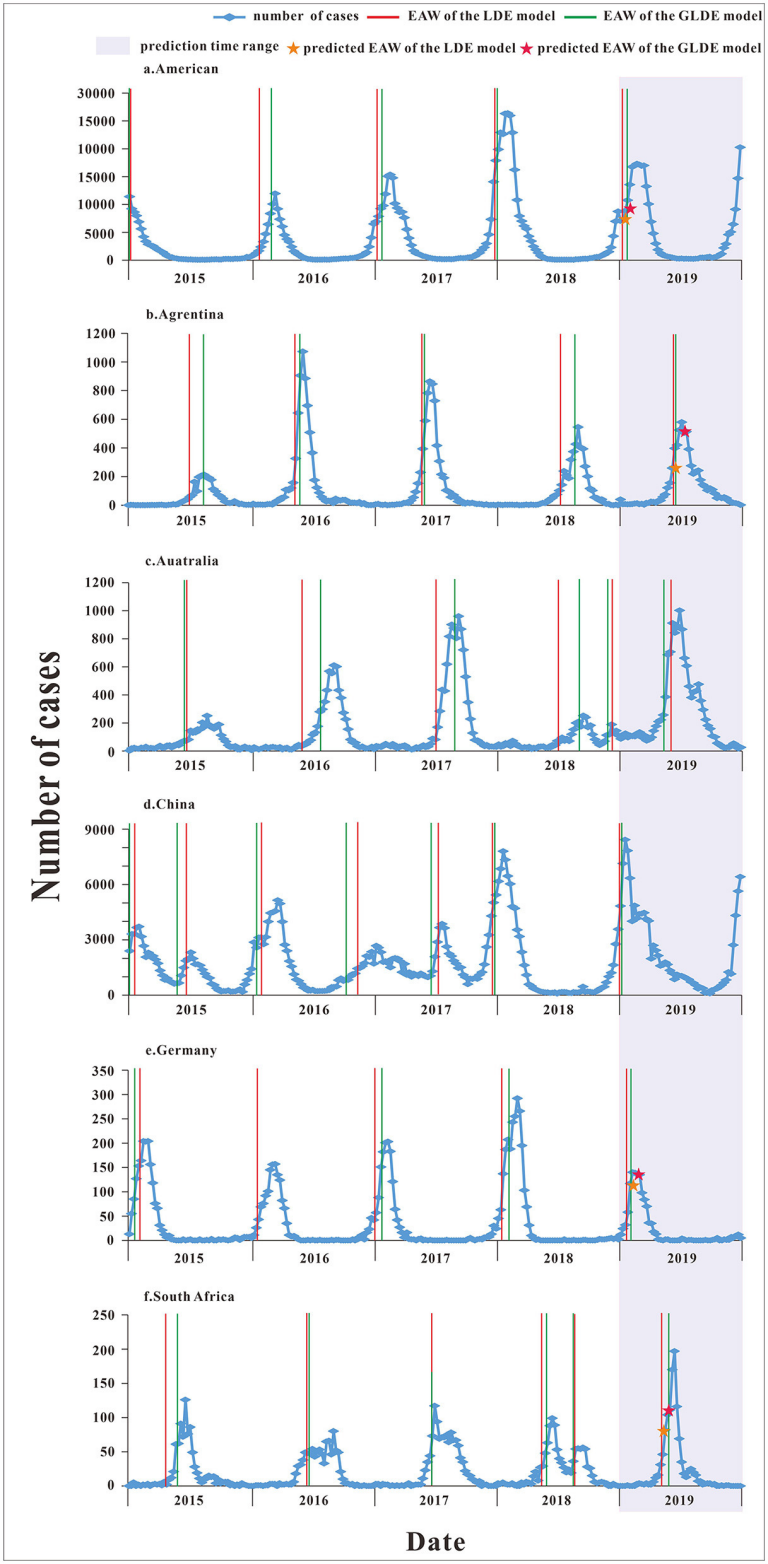
The epidemic acceleration week of logistic and generalized logistic differential equation models in different areas. **(a)** American, **(b)** Argentina, **(c)** Australia, **(d)** China, **(e)** Germany, and **(f)** South Africa.

The parameters were plugged into Equations 5, 8 to calculate each country's annual epidemic acceleration week. The last segment data of the United States and China in 2019 were not a complete wave, and it was not possible to calculate the warning week, so it was omitted. The epidemic acceleration weeks of influenza in each country are shown in Figure 7. There was only one incidence peak (in winter) in two countries (United States and Germany). There was only one incidence peak (in summer) in two countries (Argentina and South Africa), and there was also a small peak (in autumn) in South Africa in 2018. There was only one incidence peak (in autumn) in Australia, and there was also a small peak (in winter) in 2018. In addition to the winter peak, there was also a summer peak in 2015 and 2017 in China, and there was only one winter peak in the other years. The parametric test results showed that the calculated *t-*test statistic *was* −0.236 with a corresponding *p-value* of 0.025, and the difference was statistically significant, rejecting the assumption that the EAW calculated by the LDE and GLDE models was the same. So, it can be considered that EAW calculated by the LDE model was earlier than the GLDE model.

According to the corresponding median from 2015 to 2018, the epidemic acceleration time in 2019 was predicted. Because the data were divided according to the epidemic curve of the disease, the specific starting time of the epidemic in each year was different, which may affect the prediction of the epidemic accelerate week, so we excluded prediction values that were not in the actual year. For example, in Australia, the number of cases started to increase later in 2019 late than in other years; and in China, the number of cases began to increase in late 2018 and reached relatively high in early 2019, and these two predicted values differed greatly from the actual values, so they were omitted. The specific numerical value of the epidemic accelerate week of each country are shown in Supplementary File 4.

## Discussions

### Feasibility Analysis of Models

The curves of the LDE model are sigmoidal or antisigmoidal (increasing or decreasing). When the second and third derivatives of the model equation are solved, two inflection points and one peak point can be obtained. The two inflection points divide the LDE model curve into the growth, rapid growth, and slow growth phases, which respectively correspond to the beginning, peak, and end of peak periods of the epidemic curve (29). The LDE model is suitable for symmetric data and has been widely used in various infectious disease prevention, control, and early warning studies (28, 29, 38, 39). The curves of the GLDE model are similar to those of the LDE model and can be divided into the same three periods. However, due to the introduction of shape parameters, the GLDE model is suitable for symmetric data and can be used to fit asymmetric data. Currently, some studies use the GLDE model for data fitting (38).

### Advantages and Disadvantages Analysis of the Models

From the perspective of model derivation, in theory, both models can be used to predict the development trend of infectious diseases, but the applicable conditions are different and there are advantages and disadvantages in the fitting of epidemic data. The LDE model is simple, understandable, and the second derivative can be used to determine the early warning time. However, it has several disadvantages, for example, the model's inflexibility, poor fit of asymmetric or fine-tailed distribution data, and unsuitability for long-term prediction. The GLDE model can be used for epidemic data of different distribution types by introducing shape parameters, but the calculation of epidemic inflection points is more complex. The comparison of the LDE and GLDE models is shown in Table 4.

**Table 4:** Comparison of logistic differential equation (LDE) and generalized logistic differential equation (GLDE).

	**LDE**	**GLDE**
Similarity	a) Both are sigmoidal or antisigmoidal (increasing or decreasing); c) The curve can be divided into the growth, rapid growth, and slow growth phases; d) Both can be used to predict the development trend of infectious diseases; e) Both can be used to determine the early warning time; f)Neither model fitted well for scattered data (flat data with no obvious peaks); g) Both are based on epidemiological data analysis without considering the characteristics and the climatic condition of the disease.	
Application condition	Data satisfying symmetry distribution	Data of various distribution types
Input	Cumulative cases	Cumulative cases
Formula	d/dt(n) = r*n*(1-n/N);	d/dt(n) = r*n*(1-(n/N) **λ)/l;
Output	r,N	r,N,λ
Advantages	a) Simple; b) Understandable.	a) Suitable for fitting long-term epidemic data; b) Greater flexibility; c) Better fitting accuracy.
Disadvantages	a) Inflexible; b) Poor fit to asymmetric or fine-tailed distribution data; c) Not suitable for long-term forecasting.	a) More complicated to calculate of epidemic inflection points; b) May be an overfitting problem.

### The Difference of Model Fitting Results

Both LDE and GLDE models fitted the epidemic curve of the COVID-19 well, and correlations between fitting data and actual data were found (*R*^2^> 0.9, *p* < 0.05), and the goodness-of- fit of the GLDE model was better than that of the LDE model. The COVID-19 epidemic curve was asymmetrical, and the GLDE model added shape parameters to make it more suitable for fitting such data.

Observing the epidemic curves of infectious diseases with different transmission routes, we found that the regularity of the epidemic curve of influenza in respiratory infectious diseases was not distinct. There were many abnormal values and abnormal peaks. In enteric diseases, the peaks of HFMD were relatively uniform and had a notable regularity, with double seasonal peaks in spring and early winter (40–43). Among contagious infectious diseases, the AHC peak was also slightly different from the traditional peak.

There were significant outliers in A (H1N1) seasonal 2006, A (H1N1pdm09) 2009, and the total number of influenza cases in 2006, B (Yamagata lineage), 2010, and 2011. Only the outlier segmentation point of B (Yamagata lineage) in 2011 was used, and the other outliers were at their peak. The goodness-of-fit test showed that both methods could be used to data fit the outliers and, except for the LDE model in the form of B (Yamagata lineage) in 2011, the GLDE model fitted the remaining exceptions better. The derivation process of the LDE and GLDE models indicates that the models are highly stable. Individual values do not affect the overall shape of the disease epidemic curve due to long-term dispersal fluctuations. Therefore, the appearance of individual outliers does not affect the fit. Due to shape parameters being introduced, the GLDE may better fit the emergent outliers.

For data with a bifurcated peak, if it was not obvious, we ignored the bifurcation point and directly fit the entire segment, such as the thirteenth segment of A (H1N1) seasonal data. If it was obvious, we used the bifurcation point to segment, such as in the sixth and seventh segments of A (H1N1). The test results confirmed that with the two methods, the data could be fitted, but the GLDE model fitted better. This may be because the GLDE model introduces shape parameters that can be used for irregular data fitting.

In addition, there were many flat data with no obvious peaks in the influenza data, and the results of the goodness-of-fit tests showed that neither model fitted well for such scattered data. The sixth segment data of A (H1N1) seasonal cannot be fitted with the GLDE model, and the 8th and 10th segments data of B (Yamagata lineage) cannot be fitted with the LDE model. This may be because the assumption provided by the LDE model was that the data should be continuous and the data characteristics should be distributed. The “0” value often appeared in scattered data; therefore, the LDE and GLDE models were less commonly used for fitting scattered data. This is why outbreak, epidemic, and pandemic data were chosen when fitting data from different epidemic intensities. The test results also showed that the GLDE fitted well. The epidemic curve showed that the data of the three epidemic intensities were asymmetrically distributed and that the model better fitted asymmetrical distribution data after introducing the shape parameters.

When the same data were integrated into different time scales, the fitting results showed that both models produced the best goodness-of-fit using monthly data, rather than weekly and daily data. When observing the epidemic curve, we found that the monthly data distribution was smoother, which indicated that the data distribution somewhat affected the fitting. In addition, the GLDE model fitted better than the LDE model at different time scales. We speculate that the GLDE is more suitable for data with flat fluctuations after the introduction of the shape parameters.

When fitting cases data in different countries, the GLDE model fitted better than the LDE model. The pattern of the epidemic curves of influenza differed among countries, which was associated with different climatic environments, geographical conditions, population density, different traditions, and preventive and control measures (23).

In summary, although the logistic model is simple and rough, it accurately fits the disease data and describes the epidemic dynamics. The LDE and GLDE models can be used to fit epidemic data at different levels. The GLDE model fits the actual data more accurately than the LDE model, and the same result can be seen in Pelinovsky's research (23). The approximation accuracy increased significantly using the GLDE model than the LDE model, including the data for which the simple LDE model was not suitable. Studies (44, 45) also found that the introduction of the shape parameter within the GLDE model significantly improved the adjustment compared with the LDE model. Due to the significant contribution of the shape parameter, the GLDE model can not only better approximate the dynamics of data but can also capture the real data better. As the LDE model has limitations regarding the requirements for a skewed data distribution, in addition, over a long period, the parameters are not stable due to the interference factors changing, which render the LDE model unsuitable for fitting long-term epidemic data. The GLDE model is more suitable for fitting various distribution data and has better goodness-of-fit than LDE model, and this improvement is partly due to the additional parameter based on the LDE model. In fact, it is the result of the general statistical rule that the increase of the number of approximate curve parameters leads to the increase of the coefficient of determination (23). Therefore, the GLDE model may have the problem of overfitting. In practical application, we need to select an appropriate model based on the specific distribution of data. For asymmetrically distributed disease data, the GLDE model demonstrates greater flexibility and better fitting accuracy.

### The Difference of Determination of the Early Warning Week

The epidemic acceleration time of COVID-19 in Wuhan city calculated by the LDE and GLDE models was January 23 and January 24, respectively. The actual time of lockdown of Wuhan was January 23, and the calculation result of the LDE model was closer to reality. The LDE model can accurately calculate the epidemic accelerate time and then determine the critical time of the epidemic, which has important reference significance for relevant departments to take prevention and control measures, especially in major public health events.

Both LDE and GLDE models can well characterize the patterns of influenza epidemics in different countries and calculate the annual acceleration weeks. In general, The EAW calculated by the LDE model is slightly earlier than that calculated by the GLDE model. The advantages and disadvantages of the LDE and GLDE models in early warning need further study.

The fitting principle of logistic models is simple, and the calculation efficiency is high (46). The current COVID-19 pandemic is a scenario for such models that are of obvious significance (44). It can provide a rapid and timely description of epidemic dynamics in the early stage, using simple indicators, when the epidemiological characteristics of the diseases are unclear (2). When other factors become widespread, such as the difference between public health interventions and input cases abroad, the transmission of infectious diseases may complicate the research methods, and the logistic models may not be enough (47). However, it is still an important tool in modeling epidemics as it accurately estimates the critical points of the epidemic, allowing public health workers and managers to better understand the whole process of epidemic development and formulate scientific prevention and control measures (48).

Combined with the actual data of COVID-19 data and other infectious diseases, a comparative study was conducted using the LDE and GLDE models for infectious disease epidemic simulation and early warning. The scope of our study included infectious diseases with different transmission routes (respiratory tract transmission, enteric transmission, and contact transmission), different epidemic intensities (outbreak, epidemic, and pandemic), different time scales (day, week, and month), and different areas. Using *R*^2^, an indicator that reflects the goodness-of-fit, we found that the GLDE model provided a more accurate fit of data than the LDE model. Through the parametric test, we found that the LDE model provided earlier early warning week than the GLDE model. Therefore, we have reason to believe that the GLDE model is more advantageous in asymmetric infectious disease data simulation, however, its advantages of quantification in the middle and late stages of the epidemic, prediction, and early warning need to be further studied. In practice, we can choose appropriate models for disease prediction and early warning according to the region and the severity of the disease.

## Conclusion

The GLDE model provides more accurate goodness-of-fit to the data than the LDE model. The GLDE model is able to handle asymmetric data by introducing shape parameters that allow it to fit data with various distributions. The LDE model provides the earlier epidemic acceleration week than the GLDE model. We conclude that the GLDE model is more advantageous in asymmetric infectious disease data simulation.

## Data Availability Statement

The datasets used and analyzed during the current study are available from Zhuoyang Li (805493929@qq.com) on reasonable request.

## Author Contributions

YS, BZ, HZ, JL, KL, Y-CC, and TC made substantial contributions to conception and design and critically revised the manuscript for important intellectual content. YB, HZ, JL, and KL collected data. ZL, SL, JR, YB, YS, BZ, HZ, JL, KL, Y-CC, and TC conceived the experiments. ZL, SL, JR, YB, BD, QC, YZ, LL, SY, WL, and SZ conducted experiments and analyzed results. ZL, SL, JR, and YB wrote the manuscript. All authors read and approved the final manuscript.

## Funding

This study was supported by the Bill & Melinda Gates Foundation (INV-005834). The funder had no role in study design, data collection and analysis, decision to publish, or preparation of the manuscript.

## Conflict of Interest

The authors declare that the research was conducted in the absence of any commercial or financial relationships that could be construed as a potential conflict of interest.

## Publisher's Note

All claims expressed in this article are solely those of the authors and do not necessarily represent those of their affiliated organizations, or those of the publisher, the editors and the reviewers. Any product that may be evaluated in this article, or claim that may be made by its manufacturer, is not guaranteed or endorsed by the publisher.

## Abbreviations

LDE: logistic differential equation
GLDE: generalized logistic differential equation
COVID-19: coronavirus disease 2019
SARS: severe acute respiratory syndrome
A/H1N1: influenza A virus subtype H1N1
EVD: Ebola virus disease
SIR: susceptible-infective-removed
SEIAR: Susceptible-exposed-infective-asymptomatic-removed
HFMD: hand, foot, and mouth disease
AHC: acute hemorrhagic conjunctivitis
CDC: The Center for Disease Control and Prevention
WHO: World Health Organization
LRMS: the least root mean square
R^2^: the coefficient determination.
