# Supplementary material for: An Easy-to-Use Public Health-Driven Method (the Generalized Logistic Differential Equation Model) Accurately Simulated COVID-19 Epidemic in Wuhan and Correctly Determined the Early Warning Time
Source: Front Public Health. 2022 Mar 7;10:813860. doi: 10.3389/fpubh.2022.813860 (PMC8936678; doi:10.3389/fpubh.2022.813860)
Supplement: Supplementary file 1 [file Table_1.DOCX]

LDE

STARTTIME = 0

STOPTIME=105

DT = 0.02

d/dt(n)=r*n*(1-n/m);

init (n)= 1;

r 0.182022

m 49371.9

Note: Capital letters can not be recognized by Berkeley Madonna, so use m instead N.

GLDE

STARTTIME = 0

STOPTIME=105

DT = 0.02

d/dt (n) =r*n*(1-(n/m)**l)/l;

init (n)= 1;

r=0.1;

m=29971.1;

l = 0.168231

r 0.161123

m 49795.3

l 0.866989

Note: Capital letters and Greek letters can not be recognized by Berkeley Madonna, so use m instead N and l instead of λ.
