# Supplementary material for: An Easy-to-Use Public Health-Driven Method (the Generalized Logistic Differential Equation Model) Accurately Simulated COVID-19 Epidemic in Wuhan and Correctly Determined the Early Warning Time
Source: Front Public Health. 2022 Mar 7;10:813860. doi: 10.3389/fpubh.2022.813860 (PMC8936678; doi:10.3389/fpubh.2022.813860)
Supplement: Supplementary file 2 [file Data_Sheet_1.DOCX]

Table 1 Model parameter value

|  | |  | *n* | LDE | | GLDE | | |
| --- | --- | --- | --- | --- | --- | --- | --- | --- |
|  |  |  |  | *r* | *N* | *r* | *λ* | *N* |
| COVID-19 | | 1 | 1 | 0.182 | 49371 | 0.161 | 49795 | 0.867 |
| Influenza A | H1N1 seasonal | 1 | 6 | 0.268 | 1152 | 0.129 | 1243 | 0.198 |
|  |  | 2 | 1 | 0.386 | 79 | 0.287 | 82 | 0.651 |
|  |  | 3 | 1 | / | / | / | / | / |
|  |  | 4 | 1 | 0.900 | 24 | 0.312 | 25 | -0.490 |
|  |  | 5 | 1 | 0.403 | 319 | 0.190 | 360 | 0.245 |
|  |  | 6 | 1 | 0.425 | 2263 | 0.241 | 2641 | 0.469 |
|  |  | 7 | 61 | 0.617 | 1037 | 0.194 | 1426 | -0.311 |
|  |  | 8 | 24 | 0.422 | 2014 | 0.515 | 1993 | 1.318 |
|  |  | 9 | 6 | 0.465 | 748 | 0.513 | 741 | 1.144 |
|  |  | 10 | 2 | 0.186 | 90 | 0.090 | 113 | 0.207 |
|  |  | 11 | 1 | 0.605 | 753 | 0.508 | 761 | 0.790 |
|  |  | 12 | 4 | 0.765 | 867 | 0.613 | 890 | 0.726 |
|  |  | 13 | 11 | 0.329 | 4329 | 0.216 | 4531 | 0.523 |
|  |  | 14 | 6 | 0.539 | 2816 | 0.204 | 2867 | 0.013 |
|  | H1N1pdm09 | 1 | 1 | 0.563 | 32113 | 0.234 | 48622 | 0.328 |
|  |  | 2 | 1097 | 0.556 | 41586 | 0.288 | 42603 | 0.076 |
|  |  | 3 | 27 | 0.335 | 1042 | 0.160 | 1189 | 0.112 |
|  |  | 4 | 5 | 0.663 | 10673 | 0.57 | 10757 | 0.779 |
|  |  | 5 | 3 | 0.120 | 269 | 8.86e-04 | 11008 | -0.766 |
|  |  | 6 | 1 | 0.472 | 9442 | 0.164 | 10221 | 0.136 |
|  |  | 7 | 21 | 0.438 | 21901 | 0.344 | 22122 | 0.720 |
|  |  | 8 | 1 | 0.236 | 724 | 0.092 | 801 | 0.108 |
|  | H3N2 | 9 | 1 | 0.270 | 128 | 0.093 | 150 | -0.084 |
|  |  | 10 | 1 | 0.294 | 1656 | 0.101 | 1883 | 0.070 |
|  |  | 11 | 1 | 0.509 | 1147 | 0.305 | 1296 | 0.493 |
|  |  | 12 | 2 | 0.346 | 463 | 0.105 | 563 | -0.130 |
|  |  | 13 | 1 | 0.627 | 1910 | 0.171 | 2798 | 0.012 |
|  |  | 14 | 77 | 0.359 | 2733 | 0.271 | 2801 | 0.582 |
|  |  | 15 | 2 | 0.303 | 4679 | 0.384 | 4568 | 1.322 |
|  |  | 16 | 1 | 0.301 | 234 | 0.143 | 253 | 0.200 |
|  |  | 17 | 1 | 0.255 | 5313 | 1.244 | 2613 | 5.061 |
|  |  | 18 | 129 | 0.865 | 2085 | 0.272 | 2328 | -0.631 |
|  |  | 19 | 22 | 0.726 | 2107 | 0.820 | 2083 | 1.183 |
|  |  | 20 | 11 | 0.181 | 1478 | 1.66e-04 | 6.51e+09 | -0.319 |
|  |  | 21 | 12 | 0.280 | 1346 | 0.415 | 1273 | 1.630 |
|  |  | 22 | 5 | 0328 | 895 | 0.338 | 888 | 1.038 |
|  |  | 23 | 16 | 0.377 | 16162 | 0.337 | 16211 | 0.865 |
|  |  | 24 | 2 | 0.588 | 9557 | 0.333 | 10.437 | 0.463 |
|  |  | 25 | 162 | 0.591 | 2688 | 0.289 | 2751 | -0.176 |
|  |  | 26 | 1 | 0.237 | 11101 | 3.54e-04 | 1.24e+13 | -0.188 |
|  |  | 27 | 448 | 0.678 | 5934 | 0.438 | 6559 | 0.364 |
|  |  | 28 | 444 | 0.526 | 5999 | 0.265 | 6539 | -0.048 |
|  |  | 29 | 41 | 0.369 | 5806 | 0.226 | 6079 | 0.420 |
|  |  | 30 | 22 | 0.377 | 4215 | 0.142 | 6448 | 0.147 |
|  |  | 31 | 181 | 0.425 | 12674 | 0.259 | 13330 | 0.378 |
|  |  | 32 | 101 | 0.395 | 15236 | 0.240 | 16387 | 0.429 |
|  |  | 33 | 192 | 0.432 | 32131 | 0.233 | 34343 | 0.307 |
|  |  | 34 | 261 | 0.476 | 20768 | 0.268 | 21705 | 0.287 |
|  | Total | 1 | 11 | 0.248 | 1372 | 0.119 | 1516 | 0.181 |
|  |  | 2 | 1 | 0.309 | 1799 | 0.079 | 2322 | -0.078 |
|  |  | 3 | 3 | 0.300 | 1482 | 0.599 | 1330 | 2.193 |
|  |  | 4 | 6 | 0.361 | 601 | 0.077 | 851 | -0.408 |
|  |  | 5 | 3 | 0.426 | 2329 | 0.204 | 2842 | 0.321 |
|  |  | 6 | 71 | 0.427 | 2654 | 0.282 | 2744 | 0.406 |
|  |  | 7 | 2 | 0.310 | 5287 | 0.320 | 5267 | 1.040 |
|  |  | 8 | 3 | 0.468 | 3152 | 0.098 | 4288 | -0.206 |
|  |  | 9 | 35 | 0.468 | 2101 | 0.583 | 2059 | 1.354 |
|  |  | 10 | 19 | 0.263 | 5634 | 0.914 | 3528 | 3.748 |
|  |  | 11 | 149 | 0.857 | 2285 | 0.268 | 2553 | -0.651 |
|  |  | 12 | 29 | 0.680 | 2291 | 0.684 | 2291 | 1.009 |
|  |  | 13 | 15 | 0.233 | 2263 | 0.469 | 2062 | 2.287 |
|  |  | 14 | 16 | 0.554 | 2066 | 0.408 | 2135 | 0.619 |
|  |  | 15 | 35 | 0.359 | 5013 | 0.176 | 5807 | 0.241 |
|  |  | 16 | 50 | 0.370 | 65135 | 0.365 | 65551 | 0.983 |
|  |  | 17 | 1647 | 0.516 | 48661 | 0.261 | 49806 | 0.002 |
|  |  | 18 | 30 | 0.504 | 12615 | 0.302 | 13706 | 0.458 |
|  |  | 19 | 192 | 0.447 | 14429 | 0.470 | 14402 | 1.079 |
|  |  | 20 | 2 | 0.228 | 12460 | 4.29e-04 | 7.52e+11 | -0.209 |
|  |  | 21 | 485 | 0.818 | 5588 | 0.365 | 6688 | -0.104 |
|  |  | 22 | 476 | 0.530 | 6470 | 0.256 | 7093 | -0.098 |
|  |  | 23 | 46 | 0.304 | 14989 | 0.135 | 17412 | 0.199 |
|  |  | 24 | 170 | 0.339 | 5467 | 0.063 | 12385 | -0.350 |
|  |  | 25 | 240 | 0.41967 | 34401 | 0.285 | 35958 | 0.534 |
|  |  | 26 | 166 | 0.361 | 15755 | 0.244 | 16811 | 0.524 |
|  |  | 27 | 205 | 0.396 | 33126 | 0.240 | 35041 | 0.420 |
|  |  | 28 | 282 | 0.469 | 21045 | 0.269 | 21968 | 0.299 |
| Influenza B | Victoria lineage | 1 | 0 | / | / | / | / | / |
|  |  | 2 | 1 | 0.164 | 1754 | 0.187 | 1735 | 1.173 |
|  |  | 3 | 2 | 0.502 | 2440 | 0.191 | 2590 | 0.101 |
|  |  | 4 | 1 | 0.263 | 871 | 0.171 | 906 | 0.533 |
|  |  | 5 | 2 | 0.459 | 9045 | 0.400 | 9113 | 0.840 |
|  |  | 6 | 1 | 0.190 | 204 | 0.054 | 247 | -0.167 |
|  |  | 7 | 2 | 0.402 | 717 | 0.179 | 797 | 0.182 |
|  |  | 8 | 0 | / | / | / | / | / |
|  |  | 9 | 1 | 0.311 | 509 | 0.155 | 533 | 0.271 |
|  | Yamagata lineage | 1 | 0 | / | / | / | / | / |
|  |  | 2 | 11 | 0.360 | 234 | 0.190 | 259 | 0.114 |
|  |  | 3 | 1 | 0.303 | 180 | 0.110 | 234 | 0.048 |
|  |  | 4 | 1 | 0.363 | 200 | 0.201 | 220 | 0.357 |
|  |  | 5 | 1 | 0.304 | 1092 | 0.101 | 1226 | 0.025 |
|  |  | 6 | 1 | 0.337 | 531 | 0.023 | 1249 | -0.698 |
|  |  | 7 | 75 | 0.264 | 4003 | 0.632 | 3691 | 2.876 |
|  |  | 8 | 3 | 0.734 | 46 | 0.154 | 56 | -0.980 |
|  |  | 9 | 1 | 0.316 | 90 | 0.014 | 1810 | -0.413 |
|  |  | 10 | 1 | 0.511 | 163 | 0.004 | 1351 | -1.134 |
|  |  | 11 | 14 | 0.271 | 7721 | 0.188 | 7998 | 0.586 |
|  |  | 12 | 6 | 0.271 | 16359 | 0.182 | 16782 | 0.585 |
|  | Total | 1 | 10 | 0.293 | 680 | 0.070 | 839 | -0.467 |
|  |  | 2 | 1 | 0.352 | 257 | 0.377 | 255 | 1.092 |
|  |  | 3 | 1 | 0.173 | 855 | 0.350 | 836 | 2.249 |
|  |  | 4 | 1 | 0.238 | 355 | 0.241 | 354 | 1.017 |
|  |  | 5 | 1 | 0.462 | 832 | 0.241 | 880 | 0.346 |
|  |  | 6 | 5 | 0.241 | 2565 | 0.481 | 2485 | 2.240 |
|  |  | 7 | 1 | 0.230 | 1669 | 0.048 | 2745 | -0.111 |
|  |  | 8 | 19 | 0.263 | 3156 | 0.144 | 3498 | 0.332 |
|  |  | 9 | 8 | 0.501 | 221 | 0.209 | 248 | -0.117 |
|  |  | 10 | 6 | 0.271 | 1927 | 0.023 | 11973 | -0.322 |
|  |  | 11 | 5 | 0.484 | 1576 | 0.081 | 2470 | -0.404 |
|  |  | 12 | 32 | 0.479 | 21033 | 0.160 | 22384 | -0.038 |
|  |  | 13 | 45 | 0.217 | 5172 | 0.215 | 5178 | 0.992 |
|  |  | 14 | 37 | 0.198 | 26981 | 0.692 | 24956 | 3.940 |
|  |  | 15 | 9 | 0.165 | 1021 | 0.029 | 1981 | -0.340 |
|  |  | 16 | 16 | 0.271 | 22375 | 0.196 | 23041 | 0.643 |
|  |  | 17 | 47 | 0.204 | 20112 | 0.202 | 20136 | 0.981 |
| Hand, foot and mouth disease | Changsha | 1 | 70 | 0.237 | 5558 | 0.269 | 5454 | 1.191 |
|  |  | 2 | 43 | 0.417 | 2004 | 0.061 | 3101 | -0.758 |
|  |  | 3 | 23 | 0.529 | 15484 | 0.175 | 18447 | 0.019 |
|  |  | 4 | 120 | 0.361 | 3543 | 0.080 | 4996 | -0.564 |
|  |  | 5 | 10 | 0.374 | 11442 | 0.285 | 12083 | 0.706 |
|  |  | 6 | 182 | 0.381 | 903097 | 0.213 | 9689 | 0.251 |
|  |  | 7 | 48 | 0.413 | 16267 | 0.273 | 17276 | 0.539 |
|  |  | 8 | 291 | 0.322 | 8665 | 0.178 | 9585 | 0.220 |
|  |  | 9 | 64 | 0.359 | 10712 | 0.214 | 11781 | 0.428 |
|  |  | 10 | 88 | 0.349 | 9107 | 0.119 | 12602 | -0.017 |
|  |  | 11 | 122 | 0.362 | 26593 | 0.233 | 28615 | 0.504 |
|  |  | 12 | 234 | 0.420 | 6751 | 0.140 | 7672 | -0.359 |
|  |  | 13 | 52 | 0.379 | 13919 | 0.178 | 16454 | 0.253 |
|  |  | 14 | 424 | 0.551 | 4920 | 0.105 | 8607 | -0.651 |
|  |  | 15 | 370 | 0.543 | 5928 | 0.265 | 6590 | -0.027 |
|  |  | 16 | 67 | 0.375 | 21841 | 0.220 | 23792 | 0.429 |
|  |  | 17 | 214 | 0.361 | 11236 | 0.248 | 12128 | 0.527 |
|  |  | 18 | 100 | 0.279 | 15146 | 0.165 | 17968 | 0.449 |
|  |  | 19 | 337 | 0.433 | 12930 | 0.270 | 13819 | 0.376 |
|  | Xi'an | 1 | 35 | 0.300 | 8868 | 0.262 | 9062 | 0.834 |
|  |  | 2 | 109 | 0.368 | 2663 | 0.189 | 2870 | 0.063 |
|  |  | 3 | 9 | 0.563 | 20416 | 0.263 | 22237 | 0.298 |
|  |  | 4 | 188 | 0.437 | 3226 | 0.163 | 3589 | -0.409 |
|  |  | 5 | 6 | 0.406 | 8422 | 0.287 | 8947 | 0.634 |
|  |  | 6 | 104 | 0.360 | 3944 | 0.191 | 4286 | 0.171 |
|  |  | 7 | 22 | 0.411 | 17786 | 0.294 | 18606 | 0.633 |
|  |  | 8 | 299 | 0.490 | 6690 | 0.278 | 6967 | 0.13446 |
|  |  | 9 | 11 | 0.469 | 15499 | 0.237 | 16955 | 0.347 |
|  |  | 10 | 116 | 0.375 | 3591 | 0.281 | 3715 | 0.574 |
|  |  | 11 | 13 | 0.534 | 18630 | 0.253 | 20210 | 0.294 |
|  |  | 12 | 107 | 0.463 | 5405 | 0.267 | 5673 | 0.274 |
|  |  | 13 | 16 | 0.487 | 22272 | 0.267 | 23919 | 0.405 |
|  |  | 14 | 144 | 0.408 | 5701 | 0.254 | 6037 | 0.360 |
|  |  | 15 | 7 | 0.417 | 13127 | 0.302 | 13804 | 0.661 |
|  |  | 16 | 221 | 0.437 | 8264 | 0.288 | 8927 | 0.451 |
| Acute hemorrhagic conjunctivitis | | 1 | 2 | 0.621 | 337 | 0.220 | 388 | -0.053 |
| Outbreak | | 1 | 2 | 0.534 | 98 | 1.446 | 94 | 3.170 |
| Epidemic | | 1 | 0 | / | / | / | / | / |
|  |  | 2 | 1 | 0.817 | 97 | 0.320 | 109 | -0.031 |
|  |  | 3 | 1 | 0.483 | 37 | 0.015 | 127 | -1.182 |
|  |  | 4 | 3 | 0.320 | 1529 | 0.068 | 2798 | -0.135 |
|  |  | 5 | 46 | 0.556 | 3067 | 0.152 | 4090 | -0.279 |
|  |  | 6 | 55 | 0.334 | 1713 | 0.124 | 1843 | -0.326 |
| Pandemic | | 1 | 1 | 0.739 | 4950 | 0.482 | 5995 | 0.600 |
|  |  | 2 | 25 | 1.435 | 24120 | 0.241 | 38055 | -0.310 |
|  |  | 3 | 1097 | 0.575 | 40086 | 0.284 | 42810 | 0.061 |
|  |  | 4 | 71 | 0.758 | 1225 | 0.260 | 1344 | -0.497 |
| Day | | 1 | 1 | 0.079 | 514 | 7.73e-06 | 2.50e+6 | -0.811 |
|  |  | 2 | 1 | 0.058 | 951 | 0.013 | 1730 | -0.070 |
|  |  | 3 | 1 | 0.098 | 2006 | 0.019 | 2370 | -0.318 |
|  |  | 4 | 1 | 0.070 | 132 | 0.012 | 202 | -0.487 |
|  |  | 5 | 1 | 0.062 | 275 | 0.007 | 380 | -0.863 |
|  |  | 6 | 1 | 0.072 | 70 | 0.006 | 449 | -0.384 |
|  |  | 7 | 1 | 0.076 | 294 | 0.007 | 406 | -0.970 |
|  |  | 8 | 1 | 0.067 | 475 | 0.010 | 817 | -0.428 |
|  |  | 9 | 1 | 0.056 | 450 | 0.001 | 23904 | -0.411 |
|  |  | 10 | 1 | 0.070 | 787 | 0.011 | 1355 | -0.320 |
|  |  | 11 | 1 | 0.058 | 335 | 0.004 | 878 | -0.624 |
|  |  | 12 | 1 | 0.081 | 648 | 0.018 | 932 | -0.171 |
|  |  | 13 | 1 | 0.067 | 213 | 0.007 | 410 | -0.661 |
|  |  | 14 | 1 | 0.072 | 1251 | 0.013 | 1758 | -0.297 |
|  |  | 15 | 1 | 0.082 | 1839 | 0.008 | 4615 | -0.395 |
|  |  | 16 | 2 | 0.095 | 2300 | 0.017 | 2967 | -0.368 |
|  |  | 17 | 1 | 0.111 | 281 | 0.019 | 382 | -0.459 |
|  |  | 18 | 1 | 0.049 | 791 | 0.014 | 904 | -0.088 |
|  |  | 19 | 1 | 0.048 | 435 | 0.015 | 623 | 0.006 |
|  |  | 20 | 1 | 0.076 | 277 | 0.012 | 403 | -0.465 |
|  |  | 21 | 1 | 0.051 | 507 | 0.006 | 821 | -0.582 |
| Week | | 1 | 18 | 0.305 | 617 | 0.043 | 1314 | -0.542 |
|  |  | 2 | 5 | 0.356 | 979 | 0.113 | 1522 | 0.025 |
|  |  | 3 | 23 | 0.381 | 2144 | 0.155 | 2336 | -0.026 |
|  |  | 4 | 3 | 0.430 | 131 | 0.070 | 220 | -0.612 |
|  |  | 5 | 2 | 0.351 | 280 | 0.054 | 369 | -0.680 |
|  |  | 6 | 1 | 0.257 | 366 | 0.085 | 429 | -0.025 |
|  |  | 7 | 2 | 0.335 | 503 | 0.083 | 797 | -0.129 |
|  |  | 8 | 5 | 0.257 | 556 | 0.067 | 1434 | 0.006 |
|  |  | 9 | 7 | 0.350 | 853 | 0.114 | 1119 | -0.070 |
|  |  | 10 | 6 | 0.314 | 318 | 5.6e-04 | 72038 | -0.802 |
|  |  | 11 | 6 | 0.301 | 786 | 0.172 | 904 | 0.393 |
|  |  | 12 | 6 | 0.329 | 224 | 0.049 | 406 | -0.646 |
|  |  | 13 | 3 | 0.415 | 1225 | 0.085 | 1895 | -0.244 |
|  |  | 14 | 19 | 0.276 | 2283 | 0.127 | 2993 | 0.234 |
|  |  | 15 | 35 | 0.407 | 2484 | 0.140 | 2810 | -0.194 |
|  |  | 16 | 8 | 0.618 | 273 | 0.102 | 376 | -0.838 |
|  |  | 17 | 6 | 0.303 | 679 | 0.137 | 832 | 0.184 |
|  |  | 18 | 6 | 0.409 | 209 | 0.017 | 597 | -1.176 |
|  |  | 19 | 3 | 0.313 | 442 | 0.144 | 525 | 0.200 |
|  |  | 20 | 2 | 0.539 | 261 | 0.048 | 442 | -0.642 |
|  |  | 21 | 4 | 0.244 | 554 | 0.048 | 809 | -0.399 |
| Month | | 1 | 41 | 1.026 | 686 | 0.750 | 723 | 0.519 |
|  |  | 2 | 47 | 0.666 | 3271 | 0.429 | 3493 | 0.446 |
|  |  | 3 | 19 | 0.520 | 456 | 0.189 | 554 | -0.220 |
|  |  | 4 | 9 | 0.763 | 390 | 0.413 | 427 | 0.233 |
|  |  | 5 | 12 | 0.937 | 586 | 0.748 | 608 | 0.690 |
|  |  | 6 | 30 | 0.947 | 576 | 1.945 | 500 | 2.514 |
|  |  | 7 | 62 | 0.957 | 972 | 0.895 | 983 | 0.888 |
|  |  | 8 | 44 | 0.710 | 448 | 0.287 | 620 | -0.084 |
|  |  | 9 | 39 | 0.878 | 953 | 0.483 | 1007 | 0.160 |
|  |  | 10 | 22 | 1.401 | 1321 | 0.434 | 1726 | -0.192 |
|  |  | 11 | 123 | 0.732 | 2925 | 1.583 | 2367 | 2.563 |
|  |  | 12 | 308 | 0.820 | 3197 | 0.609 | 3254 | 0.440 |
|  |  | 13 | 47 | 0.778 | 869 | 0.445 | 919 | 0.169 |
|  |  | 14 | 16 | 0.784 | 696 | 0.145 | 1041 | -0.567 |
|  |  | 15 | 28 | 0.717 | 604 | 0.148 | 1042 | -0.567 |
| Influenza | American | 2015 | 11406 | 0.441 | 75262 | 1.895 | 76502 | 0.000 |
|  |  | 2016 | 91 | 0.228 | 105307 | 0.596 | 97772 | 2.882 |
|  |  | 2017 | 99 | 0.263 | 179427 | 0.348 | 176332 | 1.394 |
|  |  | 2018 | 199 | 0.333 | 294404 | 0.371 | 292988 | 1.142 |
|  |  | 2019 | 149 | 0.295 | 228793 | 0.337 | 226871 | 1.180 |
|  | Argentina | 2015 | 3 | 0.213 | 2414 | 0.562 | 2249 | 2.937 |
|  |  | 2016 | 5 | 0.382 | 6979 | 0.602 | 6904 | 1.696 |
|  |  | 2017 | 4 | 00.460 | 64778 | 0.503 | 6459 | 1.115 |
|  |  | 2018 | 4 | 0.238 | 4850 | 0.597 | 4592 | 2.783 |
|  |  | 2019 | 7 | 0.294 | 6270 | 0.282 | 6291 | 0.946 |
|  | Australia | 2015 | 3 | 0.232 | 3616 | 0.162 | 3809 | 0.615 |
|  |  | 2016 | 27 | 0.168 | 7604 | 0.749 | 6579 | 5.071 |
|  |  | 2017 | 36 | 0.171 | 12015 | 0.909 | 10309 | 6.044 |
|  |  | 2018_1 | 47 | 0.128 | 4955 | 0.027 | 26136 | 0.000 |
|  |  | 2018_2 | 53 | 0.436 | 1954 | 0.187 | 2306 | 0.000 |
|  |  | 2019 | 81 | 0.386 | 12174 | 0.204 | 12722 | 0.268 |
|  | China | 2015_1 | 2400 | 0.484 | 37679 | 0.252 | 40280 | 0.000 |
|  |  | 2015_2 | 646 | 0.475 | 23750 | 0.225 | 25010 | 0.000 |
|  |  | 2016_1 | 577 | 0.407 | 64836 | 0.212 | 68565 | 0.225 |
|  |  | 2016_2 | 227 | 0.224 | 55617 | 0.084 | 70476 | 0.089 |
|  |  | 2017 | 1047 | 0.587 | 32111 | 0.268 | 35739 | 0.000 |
|  |  | 2018 | 900 | 0.338 | 92641 | 0.297 | 93386 | 0.819 |
|  |  | 2019 | 157 | 0.204 | 109558 | 0.188 | 110404 | 0.901 |
|  | Germany | 2015 | 13 | 0.761 | 1689 | 0.387 | 1718 | 0.179 |
|  |  | 2016 | 3 | 0.243 | 1474 | 0.179 | 1406 | 4.183 |
|  |  | 2017 | 1 | 0.358 | 1552 | 0.775 | 1517 | 2.393 |
|  |  | 2018 | 3 | 0.356 | 2443 | 0.649 | 2408 | 2.022 |
|  |  | 2019 | 1 | 0.432 | 1200 | 0.519 | 1196 | 1.253 |
|  | South Africa | 2015 | 5 | 0.236 | 878 | 0.569 | 846 | 2.769 |
|  |  | 2016 | 1 | 0.276 | 992 | 0.248 | 997 | 0.866 |
|  |  | 2017 | 1 | 0.356 | 1190 | 0.264 | 1209 | 0.662 |
|  |  | 2018_1 | 5 | 0.396 | 749 | 0.546 | 712 | 1.488 |
|  |  | 2018_2 | 36 | 0.723 | 400 | 0.439 | 408 | 0.076 |
|  |  | 2019 | 1 | 0.375 | 1165 | 0.654 | 1140 | 1.895 |
