# Supplementary material for: An Easy-to-Use Public Health-Driven Method (the Generalized Logistic Differential Equation Model) Accurately Simulated COVID-19 Epidemic in Wuhan and Correctly Determined the Early Warning Time
Source: Front Public Health. 2022 Mar 7;10:813860. doi: 10.3389/fpubh.2022.813860 (PMC8936678; doi:10.3389/fpubh.2022.813860)
Supplement: Supplementary file 4 [file Data_Sheet_3.DOCX]

Table 2 Epidemic accelerate time of COVID-19 and Influenza

|  | | year | LDE | | GLDE | |
| --- | --- | --- | --- | --- | --- | --- |
|  |  |  | *t_1_* | *T_1_* | *t_1_* | *T_1_* |
| COVID-19 | | 2019-2020 | 52 | 2020.1.23 | 53 | 2020.1.24 |
| Influenza | American | 2015 | 0.920 | 1 | -0.169 | 0 |
|  |  | 2016 | 25.120 | 3 | 30.369 | 8 |
|  |  | 2017 | 23.544 | 1 | 26.245 | 3 |
|  |  | 2018 | 17.963 | 51（2017） | 19.491 | 0 |
|  |  | 2019 | 20.431 | 1 | 22.290 | 3 |
|  |  | 2019Prediction | 20.754 | 2 | 22.868 | 4 |
|  | Argentina | 2015 | 25.234 | 26 | 30.930 | 32 |
|  |  | 2016 | 15.504 | 18 | 17.899 | 20 |
|  |  | 2017 | 13.208 | 20 | 14.252 | 21 |
|  |  | 2018 | 24.328 | 27 | 29.543 | 33 |
|  |  | 2019 | 18.630 | 23 | 19.621 | 24 |
|  |  | 2019Prediction | 19.916 | 24 | 23.721 | 28 |
|  | Australia | 2015 | 24.904 | 25 | 24.088 | 24 |
|  |  | 2016 | 25.653 | 26 | 33.776 | 34 |
|  |  | 2017 | 26.286 | 26 | 34.588 | 35 |
|  |  | 2018_1 | 26.085 | 26 | 32.872 | 33 |
|  |  | 2018_2 | 5.187 | 49 | 1.947 | 47 |
|  |  | 2019 | 9.564 | 22 | 6.897 | 19 |
|  |  | 2019Prediction | 25.869 | / | 33.324 | / |
|  | China | 2015_1 | 2.832 | 3 | 0.292 | 0 |
|  |  | 2015_2 | 4.756 | 25 | 1.480 | 21 |
|  |  | 2016_1 | 8.334 | 4 | 5.596 | 2 |
|  |  | 2016_2 | 18.680 | 45 | 12.464 | 40 |
|  |  | 2017_1 | 3.531 | 27 | 1.112 | 24 |
|  |  | 2018 | 9.776 | 50 | 10.152 | 51 |
|  |  | 2019 | 25.654 | 0 | 26.838 | 1 |
|  |  | 2019Prediction | 5.933 | / | 3.354 | / |
|  | Germany | 2015 | 4.656 | 5 | 2.826 | 3 |
|  |  | 2016 | 20.057 | 2 | 130.128 | / |
|  |  | 2017 | 16.863 | 0 | 20.248 | 3 |
|  |  | 2018 | 15.134 | 2 | 18.265 | 5 |
|  |  | 2019 | 13.377 | 3 | 14.824 | 5 |
|  |  | 2019Prediction | 15.999 | 6 | 18.265 | 8 |
|  | South Africa | 2015 | 16.293 | 16 | 21.490 | 21 |
|  |  | 2016 | 20.196 | 23 | 20.817 | 24 |
|  |  | 2017 | 16.175 | 25 | 15.686 | 25 |
|  |  | 2018_1 | 9.301 | 19 | 11.012 | 21 |
|  |  | 2018_2 | 1.376 | 33 | 0.039 | 34 |
|  |  | 2019 | 15.309 | 18 | 17.948 | 21 |
|  |  | 2019Prediction | 16.234 | 19 | 18.252 | 21 |
